# A14 ABLATION OF THE INTESTINAL EPITHELIAL SIALOME PREDISPOSES TO SPONTANEOUS COLITIS AND COLITIS-ASSOCIATED TUMORIGENESIS VIA MUCUS-DEPENDENT AND -INDEPENDENT MECHANISMS IN MICE

**DOI:** 10.1093/jcag/gwad061.014

**Published:** 2024-02-14

**Authors:** K S Bergstrom, X Shan, S Rathore, L Gao, W Zandberg, K Darrek, N N, C L Letef, S Ghosh, L Xia

**Affiliations:** The University of British Columbia Okanagan, Kelowna, BC, Canada; Oklahoma Medical Research Foundation, Oklahoma City, OK; Oklahoma Medical Research Foundation, Oklahoma City, OK; Oklahoma Medical Research Foundation, Oklahoma City, OK; The University of British Columbia Okanagan, Kelowna, BC, Canada; The University of British Columbia Okanagan, Kelowna, BC, Canada; The University of British Columbia Okanagan, Kelowna, BC, Canada; The University of British Columbia Okanagan, Kelowna, BC, Canada; The University of British Columbia Okanagan, Kelowna, BC, Canada; Oklahoma Medical Research Foundation, Oklahoma City, OK

## Abstract

**Background:**

Recent work suggests epithelial sialylation (capping of glycoconjugates with the acidic sugar sialic acid) can protect the gut mucosa from inflammatory bowel disease by promoting mucus function. However, these studies are based on partial sialylation knockouts, potentially underestimating the role of glycoconjugate sialylation in mucosal homeostasis.

**Aims:**

We sought to uncover how the complete intestinal epithelial sialome (the total glycan sialylation repetoire) regulates colitis susceptibility in vivo.

**Methods:**

We generated a model of total gut epithelial sialylation deficiency by conditionally and inducibly (via tamoxifen, TM) targeting the CMP-Sia transporter, Slc35a1, in epithelial cells using Cre-Lox technology (*Slc35a1*^*f/f;*^ VillinCre(ERT2) or (TM-)IEC *Slc35a1*^*-/-*^ mice). We analyzed phenotypes clinically, histologicaly, and via multi-omic apporaches at baseline and in response to challenge.

**Results:**

We confirmed the ablation of mucus sialylation via lectin staining, epithelial *Slc35a1* gene expression, and O-glycomics of extracted mucins. In contrast to partial sialylation deficiencies, we found that IEC *Slc35a1*^-/-^ mice developed mild spontaneous microbiota-dependent chronic colitis that was associated with tumorigenesis in the rectum in 30% of aged mice (>12 mo.). TM-IEC *Slc35a1*^*-/-*^ mice developed less severe spontaneous colitis, but were susceptible to acute injury with 1% DSS. Ablation of the gut epithelial sialome led to reduced mucus thickness on fecal sections and within tissues, although the b1/b2 barrier layers were still intact. TM-IEC *Slc35a1*^-/-^ mice showed altered microbiota composition with an increase in *Clostridium* spp. at the expense of a global reduction in the abundance of at least 20 unique taxa; however metabolomic analysis did not show any significant differences in short-chain fatty acid levels. IEC *Slc35a1*^*-/-*^ Muc2 was more susceptible to fecal protease degradation vs.WT Muc2. While no spontaneous phenotype was observed in the small intestine (SI), treatment with 5-fluorouracil led to worsened SI mucositis and morbidity vs. WT littermates. This was traced to impaired stem cell activities via reduced Lgr5^+^ cell representation in small intestinal crypts in IEC *Slc35a1*^*-/-*;^Lgr5^GFP^ mice.

**Conclusions:**

Ablation of the intestinal epithelial sialome impacts the mucus-microbiota-stem cell axis with consequences on colonic mucosal homeostasis based on the type of injury. At baseline, loss of sialylation impairs mucus stability and the stem cell niche leading to microbiota-dependent spontaneous colitis and tumorigenesis. However, these same epithelial defects impair epithelial renewal and worsen colitis following acute injury likely due to the cytotoxic environment caused by the chemical agent.

**Funding Agencies:**

CCC

